# Handheld Fundus Camera for Diabetic Retinopathy Screening: A Comparison Study with Table-Top Fundus Camera in Real-Life Setting

**DOI:** 10.3390/jcm11092352

**Published:** 2022-04-22

**Authors:** Edoardo Midena, Luca Zennaro, Cristian Lapo, Tommaso Torresin, Giulia Midena, Elisabetta Pilotto, Luisa Frizziero

**Affiliations:** 1 Department of Neuroscience-Ophthalmology, University of Padova, 35128 Padova, Italy; luca.zennaro.2@studenti.unipd.it (L.Z.); cristian.lapo@aopd.veneto.it (C.L.); tommaso.torresin@gmail.com (T.T.); elisabetta.pilotto@unipd.it (E.P.); lfrizziero@gmail.com (L.F.); 2 IRCCS—Fondazione Bietti, 00198 Rome, Italy; giulia.midena@fondazionebietti.it

**Keywords:** diabetic retinopathy, screening, fundus photography, handheld fundus camera, diabetic maculopathy, hypertensive retinopathy, diabetes mellitus, Optomed Aurora, concordance coefficient

## Abstract

The aim of the study was to validate the performance of the Optomed Aurora^®^ handheld fundus camera in diabetic retinopathy (DR) screening. Patients who were affected by diabetes mellitus and referred to the local DR screening service underwent fundus photography using a standard table-top fundus camera and the Optomed Aurora^®^ handheld fundus camera. All photos were taken by a single, previously unexperienced operator. Among 423 enrolled eyes, we found a prevalence of 3.55% and 3.31% referable cases with the Aurora^®^ and with the standard table-top fundus camera, respectively. The Aurora^®^ obtained a sensitivity of 96.9% and a specificity of 94.8% in recognizing the presence of any degree of DR, a sensitivity of 100% and a specificity of 99.8% for any degree of diabetic maculopathy (DM) and a sensitivity of 100% and specificity of 99.8% for referable cases. The overall concordance coefficient k (95% CI) was 0.889 (0.828–0.949) and 0.831 (0.658–1.004) with linear weighting for DR and DM, respectively. The presence of hypertensive retinopathy (HR) was recognized by the Aurora^®^ with a sensitivity and specificity of 100%. The Optomed Aurora^®^ handheld fundus camera proved to be effective in recognizing referable cases in a real-life DR screening setting. It showed comparable results to a standard table-top fundus camera in DR, DM and HR detection and grading. The Aurora^®^ can be integrated into telemedicine solutions and artificial intelligence services which, in addition to its portability and ease of use, make it particularly suitable for DR screening.

## 1. Introduction

Diabetes mellitus (DM) is considered to be a disease of epidemic proportions. According to the International Diabetes Federation, it currently affects 463 million people worldwide, with an expected increase to approximately 700 million people by 2045 [1,2]. Diabetic Retinopathy (DR) is one of the most significant long-term complications of DM and is the leading cause of blindness in individuals of working age (20–74 years), especially in developed countries [2]. Although patients may remain asymptomatic for a long time during the disease’s progression, sight-threatening DR (including pre-proliferative severe DR, proliferative DR and diabetic maculopathy) may develop in about 6–10% of diabetic patients, with possible irreversible visual loss [3,4,5]. Therefore, screening has assumed a growing relevance in counseling at-risk patients towards adequate care and local treatment [4,6]. Recently, and particularly since the coronavirus disease 2019 (COVID-19) spread, the development of telemedicine, artificial intelligence technologies and handheld portable devices for retinal imaging has opened new perspectives in the context of retinal diseases diagnosis [5]. In particular, the use of a portable fundus camera would enable a significant implementation of screening programs, allowing general practitioners and small peripheral centers to take fundus photos to send to reading centers. Even though several portable devices have been proposed in recent years, only a few studies have validated commercially-available handheld fundus cameras, compared with consistent gold standards, in real-life screening settings [5]. The absence of studies providing practical protocols for the application of these cameras may limit their clinical relevance. The Aurora^®^ fundus camera (Optomed, Oulu, Finland) has been proposed as an adequate tool for DR screening [7]. The aim of this study was to validate the performance of this new handheld color fundus camera, the Aurora^®^, compared to a standard table-top fundus camera for the screening of DR in real-life screening setting, and to provide a practical example of the clinical application of the camera.

## 2. Materials and Methods

### 2.1. Population

This was an observational cross-sectional study with prospective enrollment. Patients affected by type 1 and type 2 diabetes who had been referred to the local screening service at the University Hospital of Padova’s center for the management of diabetic retinopathy and ocular vascular diseases were enrolled from January to June 2021. Informed consent was obtained from all patients and the study respected the principles of the Declaration of Helsinki. The study was approved by the local Ethics Committee.

Inclusion criteria were as follows: patients were age ≥ 18 years and had a diagnosis of diabetes, based on the diagnostic criteria established in 2011 by the WHO (World Health Organization); glycated hemoglobin > 6.5% on two occasions, or glycemia ≥ 126 mg/dL after at least 8 h of fasting on two occasions, or blood glucose ≥ 200 mg/dL after 2 h of an oral glucose load to be confirmed with a fasting test, or random blood glucose ≥ 200 mg/dL in the presence of typical symptoms (polyuria, polydipsia, weight loss) [8].

Exclusion criteria were as follows: patients showed poor collaboration or, the presence of disabilities that made the procedures difficult to perform or prevented adequate patient’s collaboration for the execution of the photos; allergy to mydriatics.

### 2.2. Image Acquisition

Images were acquired using both a table-top fundus camera (AFC-230, Nidek, Gamagori, Japan) already in use at the screening service, and the Aurora^®^ handheld fundus camera. Both eyes were evaluated for each patient.

The Nidek AFC-230 is a standard table-top non-mydriatic digital fundus camera that acquires high resolution (3744 × 3744 pixels, 300 dpi) 45° color images with a minimum pupil diameter for photo acquisition of 4 mm.

The Optomed Aurora^®^ is a recently introduced portable non-mydriatic digital fundus camera that allows for the acquisition of high resolution (2368 × 1776 pixels, 300 dpi) color images with a 50° angle of view. It is equipped with manual focus, with correction from −20 to +20 diopters and auto-focus, with correction from −15 to +10 diopters auto-exposure, which allows the user to automatically adapt the brightness of the image to the patient’s eye. It also contains nine internal fixation objectives for peripheral imaging. The minimum pupil diameter for photo acquisition is 3.1 mm. In addition, it is equipped with Wi-Fi for transmitting images to personal computers and for connecting to an integrated Cloud, which allows images to be sent to an optional artificial intelligence (AI) service for image analysis.

**Figure 1:**
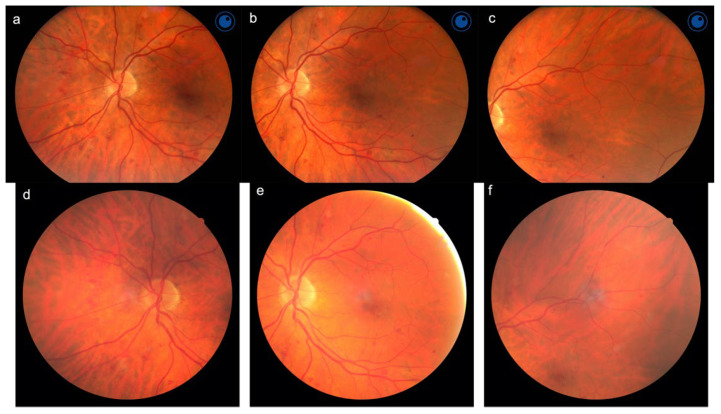
Fundus photos of the same eye affected by diabetic retinopathy, acquired using the Aurora^®^ (**a**–**c**) and the Nidek AFC-230 (**d**–**f**) fundus camera.

The image acquisition protocol consisted of three 45° (for the table-top camera) and 50° (for the handheld Aurora^®^ camera) field color images for each eye, after 1 instillation of Tropicamide 1% eyedrops: a central field, centered onto the macula; a nasal field, centered on the nasal edge of the optic disc and a superior temporal field centered temporally and superiorly to the macula, according to the currently used validated protocol [4] (Figure 1).

All images were acquired consecutively using the two fundus cameras by the same, trained, blinded operator (L.Z.) in the same environmental conditions. The operator had no prior experience in fundus imaging. Training was set to last four weeks, until the acquisition of images of three consecutive patients were judged gradable, according to two retinal specialists (E.M., E.P.).

### 2.3. Image Analysis

All images were analyzed by a single blinded operator (L.F.) in random order, using a 17-inch high-definition screen. For each eye of each patient the following parameters were assessed: gradable/ungradable, grade of DR, grade of DM, presence of hypertensive retinopathy (HR), and the presence of other diseases, for which it was deemed necessary to complete an ophthalmological examination. Images were considered as gradable when features were focused and the retinal field sufficiently illuminated and centered to allow the evaluation of any retinopathy and vessels characteristics. Furthermore, the number of photos, among the 3 acquired for each eye, that could not be evaluated due to poor quality, was reported, and the type of laser treatment identified. DR and DM grading was performed according to the International Clinical Diabetic Retinopathy and Diabetic Macular Edema Severity Scale: absent, mild, moderate, severe non-proliferative (NP) and proliferative (PDR) DR, and absent, mild, moderate and severe DM [9]. Laser-treated eyes without current signs of new vessels were defined as ex-proliferative.

Hypertensive retinopathy was considered to be present in cases of arteriolar narrowing, alterations in arteriovenous crossings (arteriovenous compression), arteriosclerosis with alterations of the vascular wall (copper wire arterioles), up to the most serious condition of hyperplasia and thickening of the vascular wall (silver wire). Eyes that were affected by severe NPDR, PDR and DM were considered as needing referral to specialistic consultation (referable) [3].

### 2.4. Statistical Analysis

The diagnostic accuracy of DR, DM and HR was assessed using sensitivity and specificity indices. The agreement between the classifications (Aurora^®^ vs. Nidek) was quantified by the proportion of observed agreement (number of eyes for which the two assessments coincided on the total number of eyes evaluated) and weighted using coefficient kappa and its 95% confidence interval. The weighing of the pairs of evaluations was conducted using a linear matrix, according to Cicchetti-Allison (CA) and a quadratic matrix, according to Fleiss-Cohen (FC) [10,11].

Since in almost all cases both eyes of the enrolled patients were evaluated, the observations were not completely independent. Therefore, the kappa statistic was considered in its variant for clustered data, where the groups (clusters) were represented by the individual patients and the observations of the group by the classifications of the right and left eyes [12].

The interpretation of the kappa value was made according to the indication of Landis and Koch: poor if kappa < 0, slight if 0–0.20, fair if 0.21–0.40, moderate if 0.41–0.60, substantial if 0.61–0.80, almost perfect if 0.81–1.00 [13,14]. All analyses were performed using SAS^®^ v. 9.4 (SAS Institute, Cary, NC, USA) on a personal computer. The SAS code macro provided by Yang and Zhou was used for the calculation of kappa [12].

## 3. Results

We included 213 diabetic patients, for a total of 423 eyes and 2538 retinal photos analyzed. The mean age of all patients was 62.6 ± 12.6 (26–85) years.

From the evaluation of images taken with the Aurora^®^, DR was detected in 110 eyes (26%) and was defined ungradable in 2 (0.47%); DM was detected in 15 eyes (3.55%) and defined ungradable in 2 (0.47%), with 15 (3.55%) eyes that needed to be referred to specialist consultation. HR was detected in 54 eyes (12.77%) and not evaluable in 1 (0.24%); other diseases were detected in 53 eyes (12.53%). There were four eyes with one ungradable photo, one eye with two ungradable photos and one eye with three ungradable photos. Focal laser treatment was found in one eye and PRP (panretinal laser photocoagulation) in two eyes (Table 1).

From the evaluation of images taken with the standard table-top fundus camera, DR was detected in 96 eyes (22.70%) and ungradable in 4 (0.95%); DM was detected in 14 eyes (3.31%) and ungradable in 4 (0.95%), with 14 (3.31%) eyes that needed to be referred to specialist consultation. HR was detected in 54 eyes (12.77%) and not evaluable in 3 (0.71%); other diseases were detected in 53 eyes (12.53%). There were four eyes with one ungradable photo, three eyes with two ungradable photos and one eye with three ungradable photos. Focal laser treatment was found in one eye and PRP (panretinal laser photocoagulation) in two eyes (Table 1).

When recognizing any degree of DR, the Aurora^®^ reached a sensitivity of 96.9% and a specificity of 94.8%, with a sensitivity of 100% and a specificity of 99.8% when recognizing referable cases. An almost perfect agreement (k 0.81–1.00) was obtained for absent, present, moderate, severe, proliferative, ex-proliferative DR and for referable cases (including DM); the agreement was instead substantial (k 0.61–0.80) for mild DR and DR gradability (Table 2). The overall concordance coefficient k (95% CI) was 0.889 (0.828–0.949) with linear weighting CA and 0.870 (0.743–0.998) with quadratic weighting FC, showing an almost perfect agreement in both cases.

When recognizing any degree of DM, the Aurora^®^ reached a sensitivity of 100% and a specificity of 99.8%. Near perfect agreement was obtained for absent, present, mild and severe DM; the agreement was instead substantial for moderate DM and DM gradability. The overall k (95% CI) was 0.831 (0.658–1.004) with linear weighting CA and 0.794 (0.544–1.044) with quadratic weighting FC, showing an almost perfect agreement in the first case and a substantial agreement in the second (Table 3).

The presence of HR was recognized by the Aurora^®^ with a sensitivity and specificity of 100%. The overall k (95% CI) was 0.960 (0.906–1.015) with linear weighting CA and 0.926 (0.827–1.025) with quadratic weighting FC, showing an almost perfect agreement in both cases (Table 4).

## 4. Discussion

Due to the dramatic increase in the global prevalence of DM, DR is a leading cause of permanent vision loss worldwide [2,15]. Since vision loss secondary to diabetes can be effectively prevented through early diagnosis and treatment of DR, its screening is an important goal in this era of diabetes “epidemic” [2]. Several local screening programs have been developed and established worldwide in recent years, most frequently as part of the pathway of care for diabetic patients. However, systematic and widespread programs are still an exception because of the lack of resources, technologies, centralized and coordinated health care systems and population information [16]. To allow its large-scale application, it is necessary to introduce new technologies to increase its convenience and feasibility. Examples include telemedicine-based programs, which allow remote image evaluation; artificial intelligence systems, for automated image analysis; and portable fundus cameras, which allow fundus photographs to be taken even outside outpatient settings, making screening much more accessible to patients [3,16,17].

Color fundus photography is currently considered the gold standard for DR screening, since both mydriatic and non-mydriatic modalities have shown a better sensitivity than direct and indirect ophthalmoscopy [16]. Screening protocols using fewer retinal fields than the traditional seven fields of the Early Treatment Diabetic Retinopathy Study (ETDRS) have proved to be equally effective in detecting referable cases, saving time and resources and increasing patients’ compliance [15]. In particular, the use of two or three standard retinal fields (central, nasal and temporal, as in our study) has shown a good agreement with the seven ETDRS fields, and are currently the most-used approaches in DR screening [15]. In most cases, current real-life DR screening consists of taking retinal photographs with a standard table-top fundus camera in dedicated, specialistic settings, followed by a manual evaluation of the images by an ophthalmologist or another specifically trained examiner. However, this approach is time-consuming and cost-intensive for both the patients and the healthcare system, and limits its large-scale use, especially in countries with scarce resources [16].

Previous studies have reported concerns about the quality of images acquired by handheld fundus cameras [18]; however, progress has been made and current image quality seems to enable adequate gradability [19]. Piyasena et al [18] showed a sensitivity of 88.7–92.5% and a specificity of 94.9–96.4% in recognizing referable cases, using the Zeiss Visuscout 100 portable fundus camera. Sengupta et al [20] found a sensitivity of 88–94% and a specificity of 84–99%, using the Optomed Smartscope portable fundus camera, while Zhang et al [21] found a sensitivity of 65–87% and a specificity of 71–90%, using the Volk Pictor Plus portable fundus camera. Kubin et al [7] reported a sensitivity of 92.3–94.2% and a specificity of 100% in recognizing referable cases, using the Aurora^®^. However, they did not consider DM and used black and white fundus photos [7]. These studies often analyzed selected populations, outside a screening setting, or using only one field or only the gradability parameter, thus limiting the applicability of the results to a real-life screening setting [18,19,20,21,22]. We applied the use of the handheld fundus camera to an established screening service, using a validated and effective imaging protocol to verify the applicability of the device, from the training of the operator to the images’ fields acquisition. In our study, the Aurora^®^ obtained a sensitivity of 100% and a specificity of 99.8% in recognizing referable cases, thus proving its effectiveness in a real-life DR screening setting, according to the British Diabetic Association guidelines [23]. Considering the possible degree of random concordance between the grading results, we also evaluated the degree of agreement (k coefficient), weighted by a linear matrix (according to Cicchetti-Allison, CA) and a quadratic matrix (according to Fleiss-Cohen, FC) [10,11]. An almost perfect linear-weighted agreement was obtained for DR, DM and HR. Three photographs of the same retinal fields were taken with both the Aurora^®^ and the Nidek camera; therefore, they both investigated the same portion of the retina. Overall, the Aurora^®^ demonstrated excellent reproducibility compared to the Nidek, which is a standard fundus camera for DR screening. The Aurora^®^, however, has the advantage of portability and ease of use, making it a superior camera in terms of investment of time and resources. Considering gradable and ungradable cases, the comparison between the Aurora^®^ and the Nidek showed a low concordance level (k). This is because the kappa statistic is sensitive to the prevalence and homogeneity of the marginal distributions of the classifications (kappa paradox) [24]. Therefore, the very low prevalence of ungradable cases made the k statistics unreliable in these cases. Conversely, the observed agreement (PO) was high (99.5%). In fact, the number of eyes with one or more ungradable images acquired with the Aurora^®^ was 1.42% (1.89% with the table-top camera). As for the other portable fundus cameras, Horus Scope DEC 200, examined by Xiao et al [22], obtained 3.2% of ungradable images in mydriasis; in the study by Zhang et al. [21] the images acquired with Volk Pictor Plus were considered gradable in 86–94% of cases in myosis and in 94–97% of cases in mydriasis; in the study by Davila et al. [19] Optomed Smartscope obtained 76.1% gradable images in miosis and 90.1% in mydriasis; in the study by Piyasena et al. [18] Zeiss Visuscout 100 obtained 70.3–76.1% evaluable images in myosis and 92.9–94.9% in mydriasis. Some authors concluded that mydriasis should be recommended at primary level, while others judged the difference between miosis and mydriasis to be clinically irrelevant [20,22]. In the screening setting, miosis would be preferable to avoid possible complications (such as needing medical staff on-site) and to make screening procedures quicker and more feasible. A study is currently ongoing to assess the performance of the Aurora^®^ in miosis.

Recently, it has also been suggested that the evaluation of retinal periphery would modify DR grading in about 10% of a selected population of diabetic patients, using widefield imaging, compared to the ETDRS 7 fields, because of a significant non-uniform distribution of DR lesions across the retina. The evaluation of retinal periphery is important in the treatment and follow-up of patients with DR, but it requires advanced and costly technologies, skills and careful evaluation. Therefore, its study is currently addressed in specialist settings, usually in second and third level centers that are suitable for the complete management of patients with advanced degrees of DR, DM or complex ocular and systemic conditions. The technologies in this field are constantly evolving and make the pathway of care for these subjects increasingly effective and accessible. However, the prevalence of DR requires a different approach in the first phases of the management of diabetic patients. Future studies will clarify the implications of peripheral lesions on DR screening, and we are currently comparing the Aurora^®^ handheld fundus camera to widefield imaging to better identify eventual differences for screening purposes.

The strengths of this study included the real-life screening setting and the use of a previously untrained photographer, adherent to our aims. The Optomed Aurora^®^ may also be integrated with an artificial intelligence service for faster retinal analysis. Future studies will evaluate the applicability of deep learning systems in health care settings [25].

Additionally, DR screening programs often include the systematic collection of data on other, non-DR ocular diseases. Retinal screening offers an opportunity to detect important conditions that would impact the care delivered to patients [26]. Therefore, the screening of other disorders has been suggested, or at least the screening of HR in DR screening. This would not only improve the correct patients’ management but also save time and resources for both patients and health care systems [26,27]. In particular, systemic hypertension is associated with an increased risk of stroke, renal impairment and cardiovascular disease, as well as the development and progression of DR. However, it is often under-recognized and undertreated [26,27]. Therefore, its detection may help patients and their treating physician to correctly address the cardiovascular risk and the whole systemic management of the condition. Several studies have proposed methods to detect HR early through the analysis of retinal vasculature [28]. The quality and the field of fundus images are fundamental for the correct HR identification and the eventual development of automatic detection programs. In our study, the Optomed Aurora^®^ proved to be effective in detecting HR and other retinal diseases compared to the standard table-top camera, thus demonstrating its suitability for screening programs that also include other retinal diseases.

## 5. Conclusions

In conclusion, we found that the Optomed Aurora^®^ portable fundus camera is effective in DR screening, since it achieved excellent sensitivity and specificity in detecting patients requiring a complete ophthalmological examination, as well as providing excellent image gradability. Thanks to its portability and simplicity in the acquisition of retinal photographs, the Aurora^®^ could also be easily used by trained technical personnel outside the outpatient setting. This, in addition to the possibility of being integrated into telemedicine solutions and artificial intelligence services, makes it particularly suitable for DR screening programs that can be convenient and sustainable even in settings with limited resources.

## Figures and Tables

**Table 1:** Distribution of diabetic retinopathy, maculopathy and hypertensive retinopathy in the enrolled population.

	Handheld Fundus Camera		Table-Top Fundus Camera	
Eyes	423		423	
Diabetic retinopathy	Freq.	Perc.	Freq.	Perc.
Absent	311	73.52	323	76.36
Mild	45	10.64	32	7.57
Moderate	61	14.42	60	14.18
Severe	1	0.24	1	0.24
Proliferative	1	0.24	1	0.24
Ex-proliferative	2	0.47	2	0.47
Ungradable	2	0.47	4	0.95
Present	110	26.00	96	22.70
Diabetic maculopathy				
Absent	406	95.98	405	95.74
Mild	9	2.13	10	2.36
Moderate	4	0.95	2	0.47
Severe	2	0.47	2	0.47
Ungradable	2	0.47	4	0.95
Present	15	3.55	14	3.31
Referable	15	3.55	14	3.31
Hypertensive retinopathy				
Absent	368	87.00	366	86.52
Present	54	12.77	54	12.77
Ungradable	1	0.24	3	0.71
Other diseases				
Absent	370	87.47	368	87.00
Present	53	12.53	53	12.53
Ungradable	0	0.00	2	0.47
Ungradable Images				
1	4		4	
2	1		3	
3	1		1	
LASER				
Focal	1		1	
PRP	2		2	

Freq: frequency; Perc: percentage; PRP: panretinal photocoagulation.

**Table 2:** The diagnostic accuracy of diabetic retinopathy grading.

	Prevalence							
Cutoff	Table-Top Fundus Camera	Handheld Fundus Camera	SE	Pos/Tot+	SP	Neg/Tot−	PO	Kappa (95% CI)
Gradability	99.0	99.5	100.0	419/419	50.0	2/4	99.5	0.665 (0.227–1.103)
Absent	76.4	73.5	94.7	306/323	95.0	95/100	94.8	0.862 (0.805–0.919)
Present	22.7	26.0	96.9	93/96	94.8	310/327	95.3	0.872 (0.816–0.928)
Mild	7.6	10.6	87.5	28/32	95.6	374/391	95.0	0.701 (0.585–0.817)
Moderate	14.2	14.4	98.3	59/60	99.4	361/363	99.3	0.971 (0.939–1.003)
Severe	0.2	0.2	100.0	1/1	100.0	422/422	100.0	1.000 (1.000–1.000)
PDR	0.2	0.1	100.0	1/1	100.0	422/422	100.0	1.000 (1.000–1.000)
Ex-PDR	0.5	0.5	100.0	2/2	100.0	421/421	100.0	1.000 (1.000–1.000)
Referable	3.3	3.5	100.0	14/14	99.8	408/409	99.8	0.964 (0.898–1.031)
Overall								
Linear (CA) Quadratic (FC)							98.6 99.4	0.889 (0.828–0.949) 0.870 (0.743–0.998)

Prevalence: The number of cases out of a total number of eyes (%); CA: Cicchetti-Allison linear weights; FC: Fleiss-Cohen quadratic weights; SE: sensitivity (%); Pos: number of positive classifications; Tot+: total number of positive cases; SP: specificity (%); Neg: number of negative classifications; Tot−: total number of negative cases; PO: observed agreement (%); Kappa: weighted kappa statistics for paired samples [12]; 95% CI: 95% confidence interval.

**Table 3:** The diagnostic accuracy of diabetic maculopathy grading.

	Prevalence							
Cutoff	Table-Top Fundus Camera	Handheld Fundus Camera	SE	Pos/Tot+	SP	Neg/Tot−	PO	Kappa (95% CI)
Gradability	99.0	99.5	100.0	419/419	50.0	2/4	99.5	0.665 (0.227–1.103)
Absent	95.7	96.0	99.7	404/405	88.9	16/18	99.3	0.911 (0.811–1.010)
Present	3.3	3.5	100.0	14/14	99.8	408/409	99.8	0.964 (0.898–1.031)
Mild	2.4	2.1	80.0	8/10	99.8	412/413	99.3	0.838 (0.627–1.050)
Moderate	0.5	0.9	100.0	2/2	99.5	419/421	99.5	0.665 (0.127–1.202)
Severe	0.5	0.5	100.0	2/2	99.5	421/421	100.0	1.000 (1.000–1.000)
Overall								
Linear (CA) Quadratic (FC)							99.3 99.5	0.831 (0.658–1.004) 0.794 (0.544–1.044)

Prevalence: The number of cases out of a total number of eyes (%); SE: sensitivity (%); Pos: number of positive classifications; Tot+: total number of positive cases; SP: specificity (%); Neg: number of negative classifications; Tot−: total number of negative cases; PO: observed agreement (%); Kappa: weighted kappa statistics for paired samples [12]; 95% CI: 95% confidence interval; CA: Cicchetti-Allison linear weights; FC: Fleiss-Cohen quadratic weights.

**Table 4:** The diagnostic accuracy of hypertensive retinopathy assessment.

	Prevalence							
Cutoff	Table-Top Fundus Camera	Handheld Fundus Camera	SE	Pos/Tot+	SP	Neg/Tot−	PO	Kappa (95% CI)
Valuability	99.3	99.8	100.0	420/420	33.3	1/3	99.5	0.498 (−0.103–1.100)
Absent	86.5	87.0	100.0	366/366	96.5	55/57	99.5	0.979 (0.951–1.008)
Present	12.8	12.8	100.0	54/54	100.0	369/369	100.0	1.000 (1.000–1.000)
Overall								
Linear (CA) Quadratic (FC)							99.5 99.5	0.960 (0.906–1.015) 0.926 (0.827–1.025)

Prevalence: The number of cases out of a total number of eyes (%); CA: Cicchetti-Allison linear weights; FC: Fleiss-Cohen quadratic weights; SE: sensitivity (%); Pos: number of positive classifications; Tot+: total number of positive cases; SP: specificity (%); Neg: number of negative classifications; Tot−: total number of negative cases; PO: observed agreement (%); Kappa: weighted kappa statistics for paired samples [12]; 95% CI: 95% confidence interval.

## Data Availability

The data presented in this study are available in the article. Eventual additional data are available on request from the corresponding author.
